# Nuclear Heparanase Regulates Chromatin Remodeling, Gene Expression and PTEN Tumor Suppressor Function

**DOI:** 10.3390/cells9092038

**Published:** 2020-09-06

**Authors:** Rada Amin, Kaushlendra Tripathi, Ralph D. Sanderson

**Affiliations:** Department of Pathology, O’Neal Comprehensive Cancer Center, University of Alabama at Birmingham, Birmingham, AL 35233, USA; ktripathi@uabmc.edu (K.T.); ralphsanderson@uabmc.edu (R.D.S.)

**Keywords:** heparanase, chromatin remodeling, gene transcription, PTEN, multiple myeloma

## Abstract

Heparanase (HPSE) is an endoglycosidase that cleaves heparan sulfate and has been shown in various cancers to promote metastasis, angiogenesis, osteolysis, and chemoresistance. Although heparanase is thought to act predominantly extracellularly or within the cytoplasm, it is also present in the nucleus, where it may function in regulating gene transcription. Using myeloma cell lines, we report here that heparanase enhances chromatin accessibility and confirm a previous report that it also upregulates the acetylation of histones. Employing the Multiple Myeloma Research Foundation CoMMpass database, we demonstrate that patients expressing high levels of heparanase display elevated expression of proteins involved in chromatin remodeling and several oncogenic factors compared to patients expressing low levels of heparanase. These signatures were consistent with the known function of heparanase in driving tumor progression. Chromatin opening and downstream target genes were abrogated by inhibition of heparanase. Enhanced levels of heparanase in myeloma cells led to a dramatic increase in phosphorylation of PTEN, an event known to stabilize PTEN, leading to its inactivity and loss of tumor suppressor function. Collectively, this study demonstrates that heparanase promotes chromatin opening and transcriptional activity, some of which likely is through its impact on diminishing PTEN tumor suppressor activity.

## 1. Introduction

Heparanase is an endo-β-D-glucuronidase capable of cleaving heparan sulfate, thereby regulating the activity of growth factors, shedding of heparan sulfate proteoglycans and degradation of extracellular matrix that together promote cell invasion, metastasis and angiogenesis by [1]. Moreover, heparanase drives exosome biogenesis and regulates exosome function, thereby controlling cell behaviors both locally within the tumor microenvironment and at sites distal to the tumor [2,3]. Heparanase is present in different subcellular locations, including within the extracellular matrix, at the cell surface and intracellularly within the cytoplasm [1]. In addition, active heparanase capable of degrading heparan sulfate is present within the nucleus and was first reported in glioma and breast cancer cells [4]. The finding that heparanase can degrade nuclear heparan sulfate is important because, within the nucleus, heparan sulfate can impact the cell cycle, cell proliferation and transcription [5]. The presence of heparanase in the nucleus leads to loss of the heparan sulfate proteoglycan syndecan-1 from the nucleus, resulting in enhanced histone acetyltransferase (HAT) activity and upregulation of expression of VEGF and MMP9, two genes that contribute to an aggressive tumor phenotype [6,7]. In activated human T lymphocytes, heparanase associates with euchromatin and regulates methylation of histone H3 that controls transcription of a cohort of inducible immune response genes [8]. Heparanase was also shown to interact with promoters of multiple genes and micro-RNAs that upregulate gene transcription and control T cell differentiation [9]. During herpes simplex virus-1 infection of corneal epithelial cells, heparanase translocates to the nucleus and enhances cytokine production [10].

In the present study, we investigated the role of heparanase in chromatin remodeling in multiple myeloma. Myeloma is characterized by the proliferation and accumulation of abnormal plasma cells within the bone marrow, causing osteolytic bone lesions, anemia, kidney failure, and poor quality of life. Despite numerous advances in therapy over the last two decades, myeloma remains difficult to control and often leads to patient mortality [11]. Therefore, mechanistic understanding of myeloma constitutes the key to identifying novel specific therapies to improve patient prognosis and outcome. Heparanase plays a major role in promoting myeloma progression, resistance to chemotherapy and maintenance of cancer cell stemness [12,13,14]. In the present work, we investigated the role of nuclear heparanase in regulating myeloma behavior. Using myeloma cell lines, we discovered that heparanase binds to nuclear chromatin and is associated with an open chromatin organization consistent with previous findings that heparanase localizes to euchromatin [8]. Heparanase enhanced the level of histone H3 within the nucleus and decreased the nuclear level of the tumor suppressor PTEN. Introduction of recombinant heparanase (rHPSE) to cells rapidly and significantly increased phospho-PTEN levels leading to stabilization of PTEN accompanied by the expected decrease in its phosphatase activity. Examination of available myeloma patient databases revealed that, when compared to patients with low heparanase expression, patients with high heparanase expression had elevated levels of histone acetyltransferase complexes, increased expression of genes associated with aggressive tumor behavior and high levels of genes that regulate the stability and activity of PTEN. Together, these findings reveal new roles of heparanase in the regulation of gene transcription in myeloma.

## 2. Materials and Methods

### 2.1. Cell Lines and Reagents

CAG human myeloma cells have been described including wild-type cells, transfectants expressing an elevated level of heparanase (HPSE) and cells in which heparanase expression was knocked down using shRNA [15,16,17] The level of heparanase in HPSE-high cells represents a physiologically relevant model for studying heparanase function because the level of heparanase in these cells is comparable to the levels displayed by some myeloma patients [15]. RPMI-8226 cells were also used as additional cells with corresponding resistance cells line for doxorubicin (RPMI-Dox-R) and resistant for melphalan (RPMI-Mel-R), provided by Dr. William S. Dalton. The resistant cell lines were developed previously by exposing cells to increased drug concentrations over time [18]. Human myeloma cell lines CAG and RPMI-8226 cells were cultured in RPMI-Glutamax media supplemented with 10% FBS, penicillin-streptomycin. 

CK2 inhibitor, 4,5,6,7-Tetrabromo-2-azabenzimidazole (TBB) was purchased from Tocris and used at 5 and 15 µM for CAG and RPMI-8226 drug-resistant cells, respectively. OGT2115 purchased from Tocris was used at 20 µM. Rabbit anti-human PTEN, phospho-PTEN, p-AKT, AKT and GAPDH were purchased from Cell Signaling, Histone 3 and acetyl-Histone 3 from Millipore, LaminB1 from Abcam and mouse anti-human PTEN and Ck2B from Santa Cruz Biotechnologies. The rabbit polyclonal anti-heparanase and recombinant heparanase (rHPSE) were a gift from Prof. Israel Vlodavsky [19].

### 2.2. MMRF Data and Gene Dataset

MMRF CoMMpass is a portal containing an extensive collection of comprehensive genomic and clinical data of myeloma patients. Gene expression data in (fragments per kilobase of exon model per million reads mapped (FPKM) were obtained from the RNA-Seq datasets AI13 “Data Availability”. These data were generated as part of the Multiple Myeloma Research Foundation Personalized Medicine Initiatives (https://research.themmrf.org and www.themmrf.org). For the study, RNA-seq from newly diagnosed patients correspond to whole bone marrow, with >50% of unfiltered CD138-positive cells (*n* = 100, IA13 data released) was used for differential gene and pathways expression. Patients were divided into two groups based on heparanase read fragment level (FPKM); patients expressing heparanase with FPKM < 0.1 (*n* = 50) were designated as HPSE-low, patients expressing heparanase with FPKM > 2 (*n* = 50) were designated as HPSE-high. 

The two independent gene datasets used for this study were from Affymetrix arrays and are publicly available at the Gene Expression Omnibus website (www.ncbi.nlm.nih.gov/geo/ accession number: GSE6477 and GSE5900). GSE6477 was generated at the Mayo Clinic from bone marrow harvested at different stages of the disease and included 15 healthy donors (HD), 22 diagnosed with monoclonal gammopathy of undetermined significance (MGUS), 24 diagnosed with smoldering multiple myeloma (SMM) and 73 with multiple myeloma (MM) [20]. In the present study, data from healthy donors and myeloma patients were utilized for the investigation of differential gene expression. GSE5900 data were generated at the University of Arkansas for Medical Sciences and derived from bone marrow plasma cells from 22 HD, 44 MGUS and 12 SMM patients [21]. In the present study, data from HD and SMM patients were utilized for investigation of differential gene expression. Ranked Gene set enrichment was performed using GSEA software (Broad Institute, San Diego, CA, USA) and identified gene set as significant with a nominal *p* value < 0.01.

### 2.3. Recombinant Heparanase and INHIBITORY Treatment

RPMI-8226 and CAG HPSE knockdown (KD) cells were treated with 1 µg/mL human recombinant heparanase (rHPSE) in serum free media at 37 °C. Treatment was ended by the addition of cold PBS followed by centrifugation at 500 rpm for 5 min. Cells were then lysed and subjected to Western blotting or cellular fractioning.

### 2.4. siRNA

Two pre-designed Silencer^TM^ siRNAs for heparanase silencing and siRNA control were purchased from Invitrogen. RPMI-8226-resistant Dox-R and Mel-R cell lines were transfected with siRNA for 48 h using RNAiMAX lipofectamine kit (Invitrogen) according to the manufacturer’s protocol. The efficiency of heparanase gene silencing was assessed by RT-PCR.

### 2.5. Chromatin, Nuclear-Cytoplasmic Fractioning

Five million cells were used for chromatin fractioning. Cells were first washed in PBS and pellets resuspended in cold lysis buffer 1 (PBS1X-1% of NP40) and immediately spun at 13,000 rpm for 10 s. The supernatant, corresponding to the cytoplasmic fraction, was transferred to a new tube. The pellet was washed once with cold lysis buffer 1 before being resuspended in low-salt buffer (10 mM Tris-HCL pH 7.5, 0.2 mM MgCl2, 0.5% Triton X100) and incubated on ice for 15 min. The mixture was spun at 13,000 rpm for 10 min and the supernatant containing the soluble nuclear fraction collected. The remaining pellet containing the chromatin fraction was resuspended in 0.2 N HCl, incubated for 20 min on ice and spun for 10 min and the pellet was discarded. The chromatin fraction was neutralized by adding an equal volume of Tris-HCl pH 7.6.

### 2.6. Chromatin Immunoprecipitation (CHIP)

Chromatin was isolated from ten million cells from CAG HPSE Hi cells and subjected to ChIP using the Simple ChIP Enzymatic Plus Kit following the manufacturer’s suggested protocol (Millipore). Bead control supplied with the kit was utilized as a negative control and chromatin containing 25 µg of DNA extract was immunoprecipitated using 5 µg of rabbit polyclonal anti-HPSE. Primers for SDC1, MMP9 and CCND1 promoter regions were purchased from Thermo-fisher. Promoter gene analysis following ChIP was performed by PCR and analyzed by electrophoresis.

MMP9 forward primer: 5′-GACAGAGCCTGGAGTGTGGGGAGG-3′, reverse primer: 5′-ACAGGCAAGTGCTGACTCAGGGGTC-3′. 

SDC1 forward primer: 5′-CCTTCCACAGCTTTTTGAACTGAGG-3′; reverse primer: 5′-TGCGCAGGACTCCTAGCTCTCTTGG-3′ 

CCND1 forward primer: 5′-CCCCGTCCTTGCATGCTAAATTAGT-3′; reverse primer: 5′-AGAGCCCAAAAGCCATCCCTGAGGC-3′.

### 2.7. Micrococcal Nuclease Assay

Nucleosome-associated DNA was assessed following the protocol from EZ Nucleosomal DNA Prep Kit from Zymo Research. After nuclei isolation using lysis buffer provided in the kit, the fraction was washed two times and incubated with 0.1U micrococcal enzyme for 11 min at room temperature. The reaction stop solution supplied in the kit was added, and DNA purified with the Qiaquick PCR Purification Kit (Qiagen) or Genejet PCR Purification Kit (Thermo Fisher) before being subjected to gel electrophoresis. 

### 2.8. Western Blotting

For lysate preparation, cells were washed and incubated in lysis buffer (50 mM Tris, pH 7.5, 150 mM NaCl, 0.5% NP-40) containing 1× HALT protease and phosphatase inhibitor mixture (Pierce) for 20 min on ice. Lysates were centrifuged at 13,000× *g* at 4 °C for 10 min, and supernatants were removed from the pellets. Equal amounts of total protein were boiled for 3 min at 95 °C before loading onto 4–20% gradient SDS-polyacrylamide gels (Bio-Rad), transferred to a nitrocellulose membrane (Schleicher & Schuell) and probed with specific primary antibodies followed by horseradish peroxidase-conjugated secondary anti-mouse or anti-rabbit antibody (GE Healthcare). Immunoreactive bands were detected using enhanced chemiluminescence (Pierce).

### 2.9. RNA Isolation and RT-qPCR

RNA was extracted from cells using TRIzol (Life Technologies), according to the manufacturer’s protocol. A total of 2 µg of RNA was then transcribed to cDNA using the High Capacity cDNA Reverse Transcription Kit (Life Technologies). A total of 20–100 ng of cDNA was used to perform RT-qPCR using Applied Biosystems Gene Expression Analysis Kit with Taqman assays (Life Technologies) according to the manufacturer’s protocol. The primers utilized were HPSE (Hs00935036_m1), SDC1 (Hs01045460_g1), MMP9 (Hs00957562_m1), VEGFA (Hs00900055_m1), CCND1 (Hs00765553_m1) and *GAPDH* (Hs02786624_g1). The real-time PCR cycle parameters and analyses were performed according to the manufacturer’s instructions.

### 2.10. Statistical Analyses

CoMMpass MMRF data were analyzed by unpaired *t*-test and gene dataset GSE were analyzed using unpaired *t*-test one tailed with Welch correction. For data in which there were three independent experiments, a one-tailed paired *t*-test was used for statistical analysis. Fold change of gene expression of MMRF data group was calculated as indicated:

$$Log2\left(FC\right)=Log2\left(\frac{\left(Average\;FPKM\;High\;group\right)}{\left(Average\;FPKM\;low\;group\right)}\right)$$

All data are presented as the mean plus or minus the standard error of the mean (SEM) and analyzed using GraphPad Prism software.

## 3. Results

### 3.1. Heparanase Enhances Chromatin Accessibility

**Figure 1:**
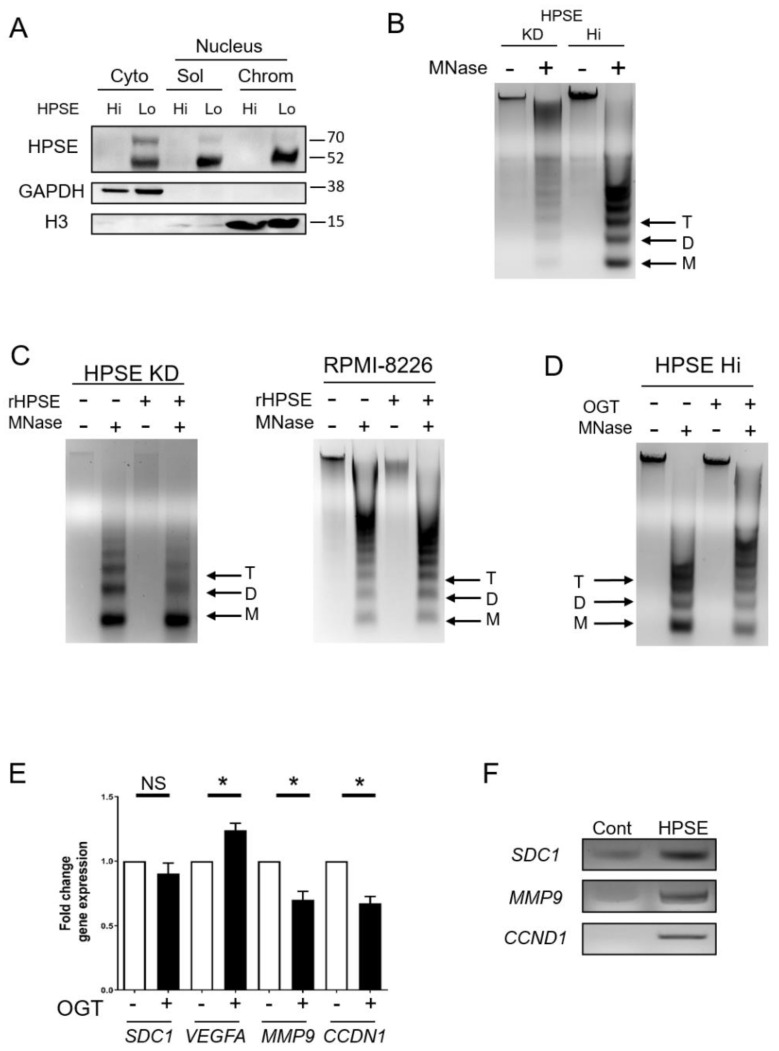
Heparanase enhances chromatin accessibility. (**A**) To determine localization of heparanase, sequential cell fractionation of myeloma cells was used to isolate cytoplasmic (cyto), nucleus-soluble (sol), and nucleus-chromatin (chrom) fractions from CAG wild-type (HPSE Lo) and transfected CAG HPSE Hi cells prior to Western blot analysis. GAPDH and histone H3 (H3) were probed to assess the quality of the fractions. (**B**) Chromatin accessibility in CAG HPSE knockdown (KD) and HPSE Hi cells were assessed by sensitivity to micrococcal nuclease (MNase) digestion. Nuclei from KD and HPSE Hi cells were prepared and digested with 0.1 U/μL MNase and subjected to electrophoresis. Black arrows denote Tri (T), Di (D) and Mono (M)—nucleosomes. (**C**) Nuclei from CAG HPSE KD and RPMI-8226 myeloma cells were treated for 2 h with or without rHPSE before being subjected to Mnase digestion for chromatin accessibility. (**D**) CAG HPSE Hi cells were incubated with 20 µM of the HPSE inhibitor OGT2115 and chromatin accessibility was assessed following exposure of nuclei to MNase. (**E**) mRNA expression analysis by RT-PCR of syndecan-1 (*SDC1*), vascular endothelial growth factor A (*VEGFA*), matrix metalloproteinase-9 (*MMP9*) and cyclin D1 (*CCDN1*) in CAG HPSE Hi cells in the presence or absence of OGT2115, compared to respective control. * *p* < 0.05 by one-tailed, paired *t*-test. (**F**) Chromatin immunoprecipitation assay was performed on CAG HPSE Hi cells to analyze HPSE binding to the promoter regions of *SDC1*, *MMP9* and *CCND1*. PCR was performed using primers probing the promoter regions of indicated genes after pulldown with an antibody control (Cont) or anti-heparanase (HPSE) and result was assessed by gel electrophoresis.

We first examined the localization of heparanase in the cytoplasm and nucleus of wild-type (HPSE Lo) or heparanase-transfected cells (HPSE Hi) of the CAG human myeloma cell line. Heparanase is only weakly expressed in wild-type cells but expressed at higher levels in the transfected cells mimicking levels seen in some myeloma tumors [15]. Sequential cell fractionation followed by Western blotting revealed heparanase in the transfected cells was present in the cytoplasm and both soluble and chromatin fractions of the nucleus (Figure 1A). This indicates an interaction of heparanase either directly or indirectly with chromatin. The 50 kDa active form of the enzyme was prevalent in all fractions, but the inactive precursor form (65 kDa) was also detected, most abundantly in the cytoplasm, but only weakly in the nucleus. To determine whether heparanase impacts the structure of chromatin, a micrococcal nuclease (MNase) assay was employed. This assay is based on MNase enzymatic digestion of linker regions between nucleosomes. An open chromatin structure is readily cleaved by the enzyme resulting in bands of small size (~200, 400 and 600 bp) corresponding to mono-, di- and tri-nucleosomes. In contrast, a closed chromatin structure is reflected by fewer small bands and higher size bands and smears. When treated with MNase, CAG myeloma cells in which heparanase had been knocked down by shRNA revealed mostly large bands of chromatin compared to smaller bands generated by MNase treatment of CAG HPSE Hi cells (Figure 1B). This indicates that when heparanase is in the nucleus, chromatin structure is open and highly accessible. To confirm this observation, nuclei were exposed to recombinant heparanase (rHPSE) and the nuclear material was assessed following MNase cleavage. Results show that nuclei exposed to rHPSE exhibited enhanced accumulation of cleaved chromatin, including most notably more of the nucleosomes of 200 bp (Figure 1C), similar to what was seen in cells expressing a high level of heparanase (Figure 1B). This result was confirmed in another myeloma cell line, RPMI-8226, in which exposure to rHPSE also enhanced the amount of smaller nucleosomes detectable following digestion with MNase. Blocking heparanase enzyme activity with the small molecule inhibitor OGT2115 resulted in a decrease in cleaved chromatin indicating that heparanase enzyme activity is required for its effect on regulating chromatin structure (Figure 1D). The open structure of chromatin that is mediated by heparanase would likely lead to enhanced gene expression. It has previously been demonstrated that upregulation of heparanase increases expression of pro-metastatic proteins including syndecan-1 (*SDC1*), MMP-9 and VEGF. Consistent with this, when CAG HPSE Hi cells were exposed to OGT2115, expression of *SDC1* and *MMP9*, as well as *CCND1* (cyclin-D1), was decreased compared to cells in which heparanase was not inhibited (Figure 1E). Moreover, Chip assays revealed that heparanase associates with the promoter regions of *SDC1*, *MMP9*, and *CCND1* (Figure 1F). To verify our heparanase ChIP assay specificity, we probed the promoter region of the *RUNX2* gene, known to promote myeloma progression [22]. Heparanase does not bind to this promoter gene, indicating that our ChIP assay does not show nonspecific pulldown (Figure S1). 

Together, these studies reveal that heparanase likely has multiple functions within the nucleus. Via its enzyme activity, it enhances chromatin accessibility and, additionally, it interacts with promoter regions of genes, potentially regulating gene transcription.

### 3.2. Elevation of Heparanase Is Associated with Increased Histone Acetylation and Upregulation of Genes that Promote an Aggressive Tumor Phenotype

**Figure 2:**
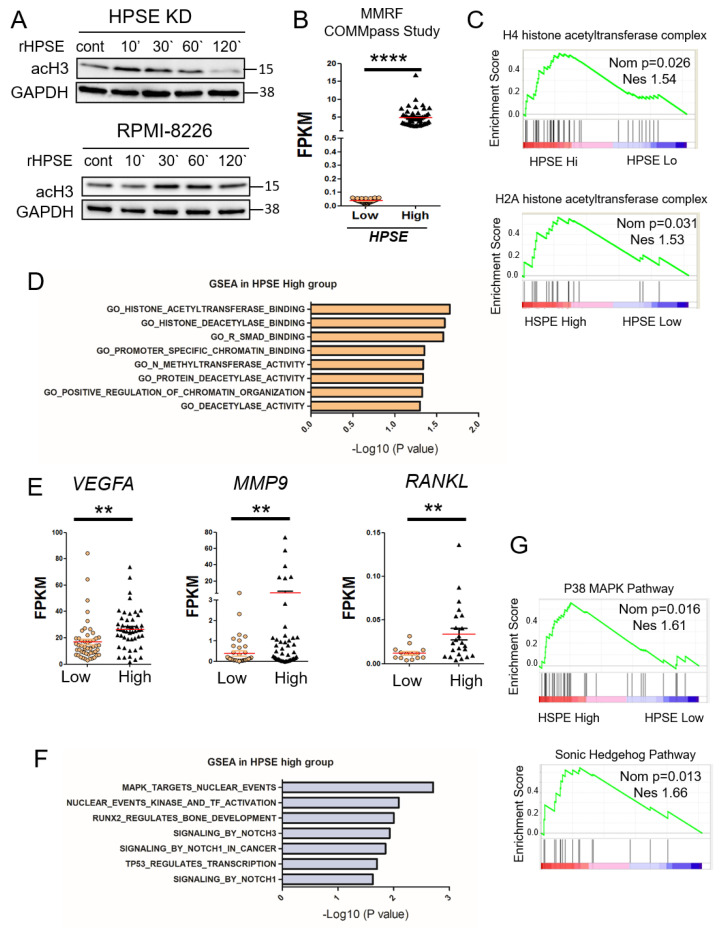
Heparanase stimulates histone acetylation and upregulation of genes that promote an aggressive tumor phenotype. (**A**) CAG HPSE KD and RPMI-8226 cells were incubated with 1 µg/mL of rHPSE for the indicated time and acetyl-histone 3 expression assessed by Western blot. (**B**) Data from the MMRF CoMMpass study were mined to assess the level of heparanase expression in a set of patients with >50% CD138+ cells. Results were plotted for patients with low heparanase (HPSE-low) expression (FPKM < 0.1; *n* = 50) and high heparanase (HPSE-high) expression (FPKM > 2, *n* = 50). The red bars represent the mean expression level of each group; **** *p* < 0.0001 by one-tailed, impaired *t*-test. (**C**) Gene set enrichment analysis (GSEA) was performed on the HPSE-high and HPSE-low patient groups, revealing enhanced acetyl-transferase signatures for H4 and H2A in the HPSE-high group. (**D**) GSEA revealed a number of genes elevated in the HPSE-high patient group that are associated with chromatin organization. (**E**) Expression levels in HPSE-low and HPSE-high groups of *VEGFA*, *MMP9* and *RANKL* mRNA, genes known to be associated with an aggressive tumor phenotype. ** *p* < 0.01 by one-tailed, unpaired *t*-test. (**F**) Top significant genes from Reactome gene sets enriched in HPSE-high patient group and ranked by -log10 *p*-value. (**G**) Biocarta gene set for p38-MAPK and Sonic-Hedgehog pathway show upregulation in HPSE-high compared to HPSE-low expressing patient tumors. Nominal *p*-value (Nom *p*) and normalized enrichment score (NES) are shown for each GSEA.

We previously demonstrated that heparanase degradation of nuclear heparan sulfate enhances histone acetyltransferase activity in myeloma cells [6]. To examine this more closely, we exposed CAG HPSE KD cells to recombinant heparanase for differing lengths of time and by Western blotting examined levels of acetylated histone H3 (acH3). Notably, exogenous heparanase trafficking in the cell has been well established to internalize within 5 min and to translocate into the nucleus after 15 min, with two-fold increases at 30 min after exposure [4,23,24]. Results demonstrated a rapid (within 10 min) increase in acH3 that diminished over the time period examined (Figure 2A). A similar, although somewhat slower response in acH3 increase was seen in RPMI-8226 myeloma cells when exposed to heparanase. To determine whether there is an association between heparanase expression, chromatin remodeling and gene transcription activity in myeloma patients, we utilized the CoMMpass dataset Al13 available from the Multiple Myeloma Research Foundation (MMRF). This dataset includes whole bone marrow isolated from 100 myeloma patients with >50% CD138-positive cells. CD138 is a widely used marker for identifying myeloma tumor cells. Patients were segregated into two groups of 50 patients, each based on heparanase relative expression: HPSE-low group in which fragments per kilobase of transcript per million mapped reads (FPKM) <0.1, and HPSE-high group in which FPKM > 2.0 (Figure 2B). The segregated high and low groups were subjected to gene set enrichment analysis (GSEA), a computational tool used to determine whether a defined set of genes with related functions shows the statistical difference between two biological states, in this case, patients expressing either low or high levels of heparanase. Patients in the HPSE-high group, as compared to the HPSE-low group, displayed elevated levels of H4 and H2A histone acetyltransferase complexes that are involved in acetylation of H4 and H2A, histones that are key components of nucleosomes (Figure 2C). These data are consistent with a role for heparanase in enhancing chromatin accessibility, as shown in Figure 1 and with previous reports [6,25]. Interestingly, GSEA also revealed that the HPSE-high patient group exhibits an elevation of three deacetylase-related genes: Genes that code for proteins that bind histone deacetylase and proteins with deacetylase activity (Figure 2D). Another signature indicating an impact of heparanase on enhancing DNA accessibility is the positive correlation with methyltransferase activity, an enzyme known to catalyze the transfer of one, two or three methyl groups onto histone proteins. Methylation of histones can either increase or silence gene transcription depending on the site of histones that have been methylated. Active methylated histone weakens their interactions with DNA, thereby promoting uncoiling of DNA from nucleosomes and enhancing accessibility of transcription factors to their target genes. Also enriched in the HPSE-high myeloma group was a set of genes encoding Smad binding proteins. This may also be related to the regulation of chromatin structure because members of the Smad family are known to be involved in chromatin histone modification and transcription activity [26]. The HPSE-low patient group does not display any chromatin signature or pathway related to active transcription (Figure S2). Further analysis confirms that the HPSE-high patient group has elevated levels of downstream target genes, including *VEGFA*, *MMP9* and *RANKL* (Figure 2E), three genes whose proteins are known to be upregulated in cells expressing a high level of heparanase. In addition, the HPSE-high group is enriched in expression of genes linked to several important signaling pathways known to drive myeloma behavior and bone homeostasis (Figure 2F,G).

### 3.3. Heparanase Causes Loss of PTEN from the Nucleus and Upregulation of Genes Associated with an Aggressive Tumor Phenotype

**Figure 3:**
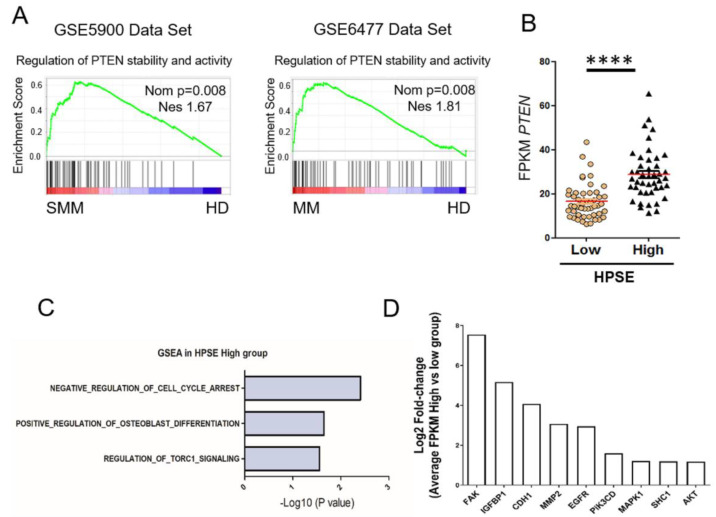
PTEN stability and activity are upregulated in myeloma. (**A**) GSEA analysis of datasets comparing healthy donors (HD) to smoldering myeloma patients (SMM) and healthy donors to multiple myeloma patients (MM). PTEN signatures were enriched in patients compared to healthy donors (red bar tracks high enrichment, blue bar tracks low enrichment of genes in the signature). Nominal *p*-value (Nom *p*) and normalized enrichment score (NES) were displayed for every GSEA analysis (MM, multiple myeloma; SMM, smoldering multiple myeloma). (**B**) Expression of PTEN in patients from the HPSE-high and HPSE-low group (as shown in Figure 2B), **** *p* < 0.0001 by one-tailed, impaired *t*-test. Comparison of myeloma tumor cells from the HPSE-high patient group versus the HPSE-low group reveals elevated expression in the HPSE-high group of gene sets (**C**) and genes (**D**) known to be correlated with downregulation of PTEN activity (log2 transformation of fold change of the relative FPKM expression in (**D**) comparing HPSE-high group with low group).

While mining patient databases probing for GSEA signatures potentially related to heparanase-mediated control of chromatin organization and gene expression, we discovered that sets of genes that regulate stability and activity of phosphatase and tensin homolog deleted on chromosome 10 (PTEN) are enriched in patients diagnosed with smoldering myeloma or myeloma (Figure 3A). This is of interest because PTEN acts as a tumor suppressor that controls chromatin organization and transcription activity of cells [27]. The mutation rate of PTEN in myeloma is low [28]; thus, there have been limited studies related to PTEN in myeloma. Only 5.6% of myeloma cases investigated displayed heterozygous mutations, which suggests other mechanisms of PTEN dysfunction may be in play. To determine whether one such mechanism is related to heparanase presence and activity in the nucleus, we first assessed whether myeloma patients in the HPSE-high group (from the MMRF CoMMpass database, Figure 2B) had elevated PTEN as compared to patients in the HPSE-low group. Results reveal there is a statistically significant higher level of PTEN in the HPSE-high group, indicating a possible association of heparanase with PTEN stability in the nucleus. Comparison of GSEA analysis of the HPSE-high and HPSE-low myeloma patient groups revealed that HPSE-high patients exhibit upregulation of biological pathways and genes known to be downregulated by PTEN (Figure 3C,D) [29,30,31,32,33,34,35,36,37].

**Figure 4:**
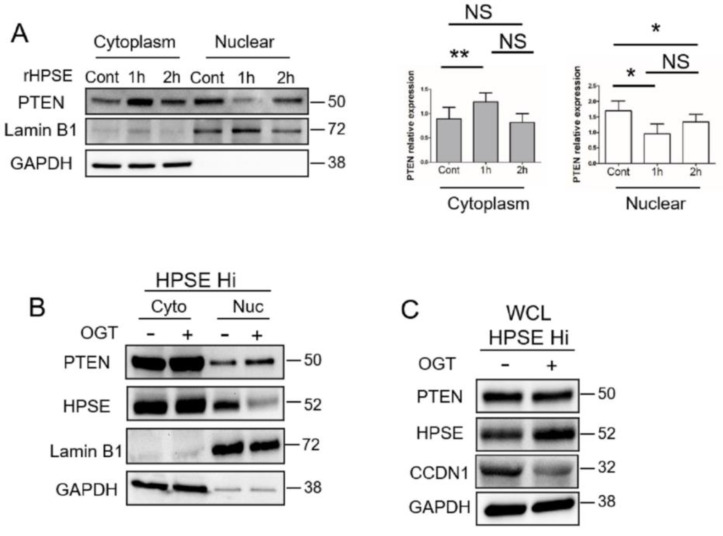
HPSE reduces nuclear PTEN level. (**A**) RPMI-8226 cells were treated with PBS as a control (Cont) or rHPSE (1 µg/mL) for the designated time followed by isolation of cytoplasmic and nuclear fractions. PTEN present in each fraction was assessed by Western blotting. LaminB1 and GAPDH were used as loading control and to assess the quality of the isolated fractions. The bar graphs show the mean relative expression of PTEN normalized with GAPDH for the cytoplasmic fraction or normalized with LaminB1 for the nuclear fraction in three independent experiments. ** *p* < 0.01 and * *p* < 0.05; NS—not significant. (**B**) CAG HPSE Hi cells were treated with OGT2115 and the levels of PTEN and HPSE present in the cytoplasmic and nuclear fractions were evaluated by Western blot. LaminB1 and GAPDH were utilized for assessing the quality of the fractions. (**C**) HPSE Hi cells were incubated without or with the heparanase inhibitor OGT2115 for 16h. PTEN, CyclinD1 (CCND1) and HPSE expression were evaluated in whole cell lysates (WCL) by Western blot.

To probe this further, RPMI-8226 myeloma cells were exposed to rHPSE, the cells fractionated, and the sub-cellular compartments assessed for PTEN expression. Following one hour of exposure to rHPSE, the nuclear PTEN level dropped dramatically, and the level of cytoplasmic PTEN increased (Figure 4A). Similarly, the PTEN level was low in the nucleus of CAG HPSE Hi cells relative to the high level of PTEN in the cytoplasm (Figure 4B). Moreover, the PTEN level in the nucleus was enhanced when the cells were exposed to the heparanase inhibitor OGT2115. Interestingly, OGT2115 exposure led to a decrease in detectable heparanase in the nucleus, raising the possibility that heparanase enzyme activity is required for its nuclear localization or stability. Next, to determine whether PTEN present in the nucleus is active following heparanase inhibition, we examined the level of CCDN1, a marker whose expression level and nuclear localization are reduced by active PTEN. Results show a reduction in the level of CCND1 following exposure of cells to OGT2115 (Figure 4C). 

Together, these data indicate that heparanase reduces levels of nuclear PTEN thereby reducing its tumor suppressive function in these cells as manifested by upregulation of genes that drive tumor growth and progression (Figure 1E and Figure 2E).

### 3.4. Heparanase Increases PTEN Expression and Phosphorylation through CK2 to Promote PTEN Protein Stability Leading to Its Inactivity

**Figure 5:**
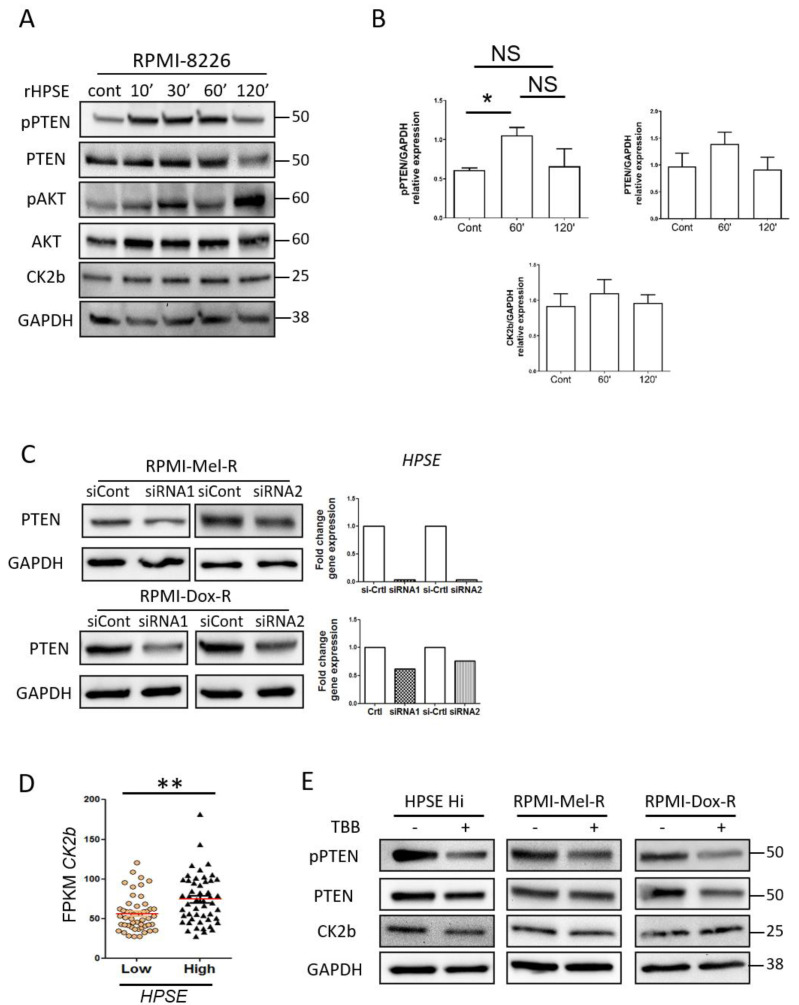
Heparanase increases PTEN phosphorylation through CK2 and promotes PTEN protein stability and inactivity. (**A**) RPMI-8226 control cells were treated with 1 ug/mL of recombinant heparanase (rHPSE) for the indicated time and pPTEN, PTEN, p-AKT, AKT and CK2b protein expression were investigated by Western blot. (**B**) Quantification of Western blot shown in panel A corresponding to the relative expression of pPTEN, PTEN and CK2b normalized with GAPDH expression, from three independent experiments. * *p* < 0.05 by one tailed Mann Whitney test. (**C**) RPMI-Mel-R and RPMI-Dox-R were transfected with siRNA control (siCont) or with two non-overlapping siRNAs to silence HPSE and PTEN level evaluated by Western blot. Right panel, *HPSE* silencing was evaluated by RT-PCR. (**D**) *CK2b* levels present in HPSE-high and HPSE-low patient groups. ** *p* < 0.01 by one-tailed, unpaired *t*-test. (**E**) CAG HPSE Hi and melphalan-resistant (Mel-R) and doxycycline-resistant (Dox-R) RMPI-8226 cell lines were incubated with CK2 inhibitor TBB for 6 h and Western blots performed to assess levels of pPTEN, PTEN and CK2b.

To further probe the impact of heparanase on PTEN in myeloma cells, we examined whether heparanase alters the phosphorylation state of PTEN. This is important because phosphorylation of PTEN maintains the protein in a stabilized state, a state in which its phosphatase activity is inhibited leading to loss of its tumor suppressive function. In the cytoplasm, PTEN lipid-phosphatase activity has been reported to be one of its important functions as tumor suppressor, because it acts as an antagonist to the PI3K/AKT signaling axis thereby regulating growth, cell proliferation, angiogenesis and cell survival [38]. Phosphorylation of PTEN occurs in a cluster of serine and threonine residues near the C-terminal tail (Ser380, Thr382, Thr383, and Ser385) resulting into a closed protein conformation and accumulation in the cytoplasm [39]. We assessed phosphorylation of PTEN at Ser380-Thr382 sites before and after exposure of RPMI-8226 cells to rHPSE. PTEN is rapidly phosphorylated (within 10 min) and maintained in this state for one hour after treatment (Figure 5A). The saturation level of phosphorylation is reached at 60 minutes and decreased after 120 minutes of exposure to rHPSE (Figure 5B). Consistent with phosphorylation of PTEN enhancing PTEN stability, the level of PTEN increases up to the 60 min time point and then is diminished after 120 min, closely paralleling the level of pPTEN. To confirm that the pPTEN has lost its functional phosphatase activity, we assessed AKT phosphorylation, a known target of the phosphatase activity of PTEN. With the exposure of cells to rHPSE, accompanied by enhanced phosphorylated PTEN, the level of pAKT increases (Figure 5A).

To further examine heparanase regulation of PTEN level, we knocked down heparanase in RPMI-8226 drug-resistant cell lines RPMI-Mel-R (melphalan resistant) and RPMI-Dox-R (doxorubicin resistant), both which express a high endogenous level of heparanase [13]. Heparanase was knocked down with either of two non-overlapping siRNA sequences and heparanase knockdown confirmed by RT-PCR (Figure 5C, right panel). Western blots demonstrate that when heparanase is knocked down, PTEN levels are diminished in both resistant cell lines. 

Several reports have demonstrated that PTEN phosphorylation at Ser380-Thr382 is regulated by casein kinase 2 (CK2) [40]. We investigated the expression of one of the subunits of CK2, CK2b, following exposure of cells to rHPSE and found that CK2b does not increase significantly, but does show a slight increase as compared to the control level (Figure 4A). When probing patient expression data, we found that as a group, the HPSE-high patients (those identified in Figure 2B) displayed more CK2b gene expression than the HPSE-low group (Figure 5D). These data suggest the possibility that a HPSE-mediated increase in CK2b contributes to PTEN phosphorylation and inactivation. Next, to determine whether CK2b was contributing to the phosphorylation of PTEN in cells expressing high heparanase, we utilized TBB, an inhibitor of CK2 activity. When cells were exposed to TBB, PTEN and CK2b levels remained stable. However, the amount of phosphorylated PTEN was markedly diminished (Figure 5E).

Together, these data demonstrate that heparanase contributes to PTEN stability by enhancing its phosphorylation, at least in part via the activity of CK2b.

## 4. Discussion

Although heparanase has been studied extensively for its role in regulating tumor invasion and metastasis in myeloma and other cancers, little is known regarding the role of heparanase within the nucleus. In the present study, using myeloma cell lines we find that: (i) within the nucleus, heparanase is present in the soluble fraction, and it is also bound to insoluble chromatin; (ii) the presence of nuclear heparanase enhances acetylation of histone H3 and promotes an open chromatin conformation; (iii) heparanase binds the promoter region of syndecan-1, MMP9 and CCND1, three genes whose expression is upregulated by heparinase; and (iv) heparanase increases phosphorylation of PTEN, leading to enhanced PTEN stability thereby diminishing its function as a tumor suppressor. Examination of available gene expression databases revealed that myeloma patients with high heparanase expression exhibited enhanced expression of acetyltransferase complexes and signaling pathways associated with myeloma growth and progression. High heparanase in myeloma patients was associated with elevated patient PTEN levels compared to patients with low heparanase, consistent with PTEN stabilization and loss of tumor suppressor activity. Additionally, both smoldering myeloma and myeloma patients displayed an increase in the genes that regulate PTEN stability and activity when compared to PTEN expression in healthy donors. Taken together, these data reveal an important role for nuclear heparanase in regulating gene transcription that helps drive the aggressive phenotype of myeloma tumors. 

Interestingly, the data indicate that heparanase enzyme activity is important for its presence in the nucleus. Very little latent heparanase is present in nuclear fractions (Figure 1A) and heparanase present in the nucleus is lost when cells expressing a high level of heparanase are treated with the heparanase inhibitor OGT2115 (Figure 4B). These findings suggest that either enzyme activity is required for translocation of heparanase to the nucleus and/or for its retention within the nucleus. OGT2115 is a small molecule inhibitor, but in the myeloma cells, it is not known whether it is blocking heparanase activity within the cytoplasm and inhibiting transport to the nucleus, or it is present in the nucleus, where it could block heparanase activity resulting in loss of nuclear heparanase. Whichever is the case, the amount of heparanase in the nucleus decreases following treatment with OGT2115 and this results in a decrease in the amount of chromatin in the open conformation (Figure 1D). Additionally, latent heparanase is activated when it is cleaved by cathepsin L [41] and studies of murine myeloma have shown that reduction in cathepsin L levels greatly reduces tumorigenesis and growth in mice [42,43], a potential link with loss of heparanase activation. 

It was previously demonstrated that nuclear syndecan-1 heparan sulfate inhibits histone acetyltransferase activity and that heparanase activity can reverse this effect by degrading nuclear heparan sulfate chains [6]. Conversely, heparanase expression by cells enhances syndecan-1 shedding from the cell surface and the shed syndecan-1 can be rapidly taken back up by cells and translocated to the nucleus, where it inhibits histone acetyltransferase activity [44]. Thus, the balance between the level of nuclear and non-nuclear heparanase and syndecan-1 shedding potentially provides a mechanism for fine-tuning the expression of multiple genes that regulate tumor behavior. This idea is further supported by the rapid increase in acetylated histone H3 that occurs within 10 min of exposure of cells to rHPSE (Figure 2A). Moreover, the finding that exogenous heparanase can enhance nuclear acetyltransferase activity indicates that tumor cells do not necessarily have to be producing heparanase for it to regulate gene expression. Thus, the level of heparanase within the tumor microenvironment that is the result of secretion of the enzyme of by other tumor cells, or by other cells known to produce heparanase (e.g., immune cells and macrophages), may play a critical role in regulating expression of genes that drive tumor progression. 

Furthermore, heparanase can also interact with additional nuclear partners to regulate histone modification and transcription activity. He et al. reported that heparanase interacted with RNA polymerase II and with an active methylated histone 3 to preferentially concentrated at the euchromatin, the active region of chromatin [8]. Heparanase not only binds to promoters but can also bind to 5′ coding regions and influence histone methylation to modulate transcription. Mechanistically, heparanase recruits the demethylase LSD1, to induce gene activation through histone demethylation, while the methylase MLL, an antagonist of LSD1 function, is recruited to the repressive complex of transcription when heparanase is knocked down [8]. Altogether, nuclear heparanase can bind to several regions of genes and regulate distinct transcription complexes.

To meet cellular needs, transcription of genes is regulated by dynamic modulation between an open and closed chromatin conformation [45]. This equilibrium between nuclear compaction and decompaction is often lost in cancer, underscoring the importance of proper regulation of nuclear compaction status [46]. Heparanase nuclear activation appears to respond cellular changes. For example, in activated T cells heparanase binds a large cohort of genes related to development and differentiation pathways while when cells were in the resting condition, heparanase bound to genes related to the metabolic and biosynthetic processes [8]. These findings indicate that nuclear heparanase function is triggered to modulate transcription in response to cellular needs.

PTEN is an important tumor suppressor that governs a plethora of biological processes depending on its subcellular localization. In the cytoplasm, the tumor suppressive role of PTEN is essentially through inhibition of the PI3K/AKT axis, where it mainly fulfills its lipid-phosphatase activity to maintain cell homeostasis. While in the nucleus, PTEN regulates the global profile of gene transcription through the maintenance of chromatin integrity. PTEN associates with histones and controls chromatin condensation [27]. Additionally, PTEN helps maintain chromosomal stability by physically interacting with centromeres and regulating DNA repair [47]. While PTEN is frequently mutated in several cancers, its rate of mutation in myeloma is low, with 5.6% of patients showing a heterozygous mutation and only observed in the advanced stage of cancer [28]. However, PTEN function can be compromised by mutation or epigenetically by transcriptional repression, microRNA, or post-translational modification (e.g., phosphorylation, ubiquitination, and oxidation). Little is known regarding the loss of PTEN function in myeloma, although miRNA or gene methylation may play a role [48,49,50]. The present study is the first to show that heparanase acts as a potential inhibitor of PTEN, by regulating its nuclear localization and its phosphatase activity. We discovered that heparanase displaced nuclear PTEN and enhanced targeted genes associated with PTEN function. Importantly, Chen et al. showed that chromatin integrity is impaired upon PTEN deletion or upon hyperacetylation of histone leading to physical dissociation of nuclear PTEN from chromatin. This indicates that a decrease in nuclear PTEN is partially due to the increase in acetylation mediated by heparanase. Additionally, we also demonstrate that heparanase can repress PTEN phosphatase activity through phosphorylation presumably via CK2 (Figure 4 and Figure 5). Consequently, loss of phosphatase activity of PTEN leads to aberrant activation of AKT, that is often associated with promoting tumor survival, resistance to apoptosis and growth [51,52]. Interestingly, heparanase has been shown to enhance levels of activated AKT and MAPK pathways [13,53]. Our results raise the possibility that at least a portion of this increase in activated AKT and MAPK may be due to loss of PTEN phosphatase activity mediated by heparanase.

Regarding the cellular signaling events that heparanase and PTEN regulate, an intriguing crosstalk between these two can be proposed. Activation of genes that are associated with high heparanase expression in myeloma patients such as Notch, Sonic Hedgehog, Smad and the Tor complex C1 (Figure 2 and Figure 3) are among the major regulators of cancer stem cell activities, and their alterations are associated with tumorigenesis in myeloma [54,55,56,57,58]. Interestingly, mTOR, Notch and Sonic Hedgehog promote self-renewal and survival of tumor-initiating cells by targeting PTEN [59,60,61,62]. While PTEN emerged to be critical for stem cell maintenance playing a role in self-renewal, and proliferation, we recently published that heparanase promotes stemness properties of myeloma by increasing stem cell-related genes and proliferation. Heparanase increases expression of GLI1, a member of the Sonic Hedgehog pathway, utilizing NF-Kb as a mediator of transcription. Interestingly, NF-Kb is a target of PTEN and knockdown of PTEN enhances stemness through AKT/NF-KB signaling in prostate cancer [63,64]. Activation of these cancer stem cell regulators suggests stem cells are initiated by increased heparanase coupled with the loss of PTEN. 

In myeloma, we have demonstrated that heparanase promotes osteolysis, angiogenesis and chemoresistance [12]. In contrast, PTEN has the opposite effect as it downregulates these phenomena in cancer. For example, heparanase increases osteolysis and bone destruction through increased expression of RANKL and MMP9, while PTEN suppresses MMP9 and RANKL-mediated osteoclast differentiation [65,66]. Furthermore, HPSE promotes cell adhesion and tumorigenesis through integrin mediator FAK and AKT, while PTEN dephosphorylates and inhibits FAK expression, suppresses AKT phosphorylation and inhibits integrin-mediated spreading and cell migration [53,67]. In myeloma, re-expressing wild-type PTEN in PTEN-deficient cells represses proliferation and invasion by downregulating FAK, MMP2 and MMP9 expression and activity [68], indicating that HPSE and PTEN are targeting expression of the same proteins but in opposite ways.

Together these findings indicate that nuclear heparanase plays an important role in tumor progression by promoting chromatin remodeling that opens its conformation allowing access to promotors of genes that drive progression. Additionally, by diminishing the phosphatase activity of PTEN, thereby blocking its function as a tumor suppressor, heparanase ensures further activity of biological pathways that enhance tumor growth, progression and resistance to therapy.

## Supplementary Materials

The following are available online at https://www.mdpi.com/2073-4409/9/9/2038/s1. Figure S1: HPSE binding to promoter region of *RUNX2* genes analysis by ChIP assay. Figure S2: Top significant genes from biological process (A) and Reactome (B) enriched in HPSE-low patient group and ranked by −log10 *p* value.
