# Supplementary material for: Nuclear Heparanase Regulates Chromatin Remodeling, Gene Expression and PTEN Tumor Suppressor Function
Source: Cells. 2020 Sep 6;9(9):2038. doi: 10.3390/cells9092038 (PMC7564302; doi:10.3390/cells9092038)
Supplement: Supplementary file 1 [file cells-09-02038-s001.pdf]

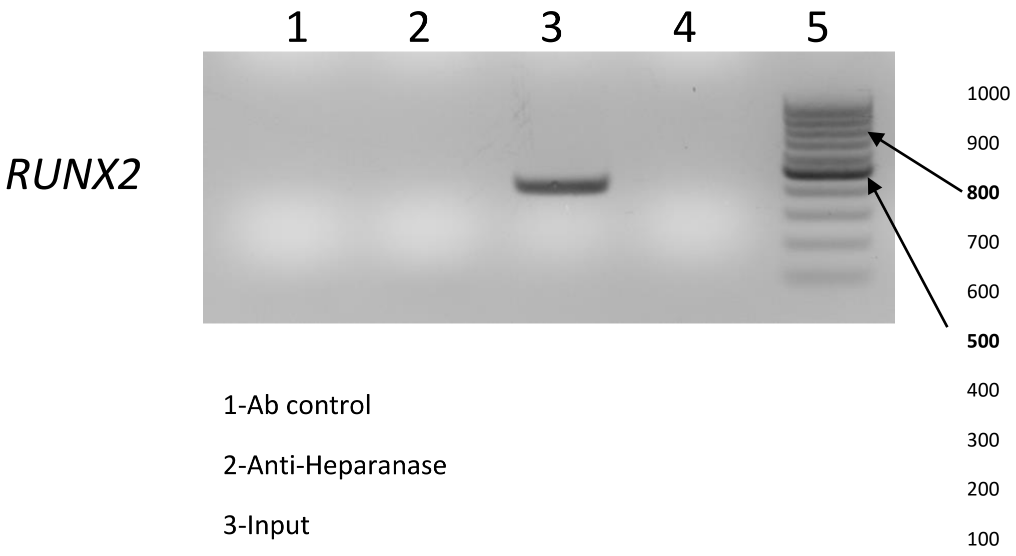

1-Ab control

2-Anti-Heparanase

3-Input

4-PCR negative control (without DNA)

5-Ladder

Figure S1: HPSE binding to promoter region of *RUNX2* gene analysis by ChIP assay. Chromatin immunoprecipitation assay was performed on CAG HPSE high cells to analyze HPSE binding to promoter region of *RUNX2*. PCR was performed using primers probing the promoter regions of *RUNX2* gene after pulldown with an antibody control (Ab, line 1) or anti-heparanase (line 2). Input (Line 3) was used as positive control of design primers for *RUNX2* promoter genes and line 4 was used a negative control of PCR quality and specificity.

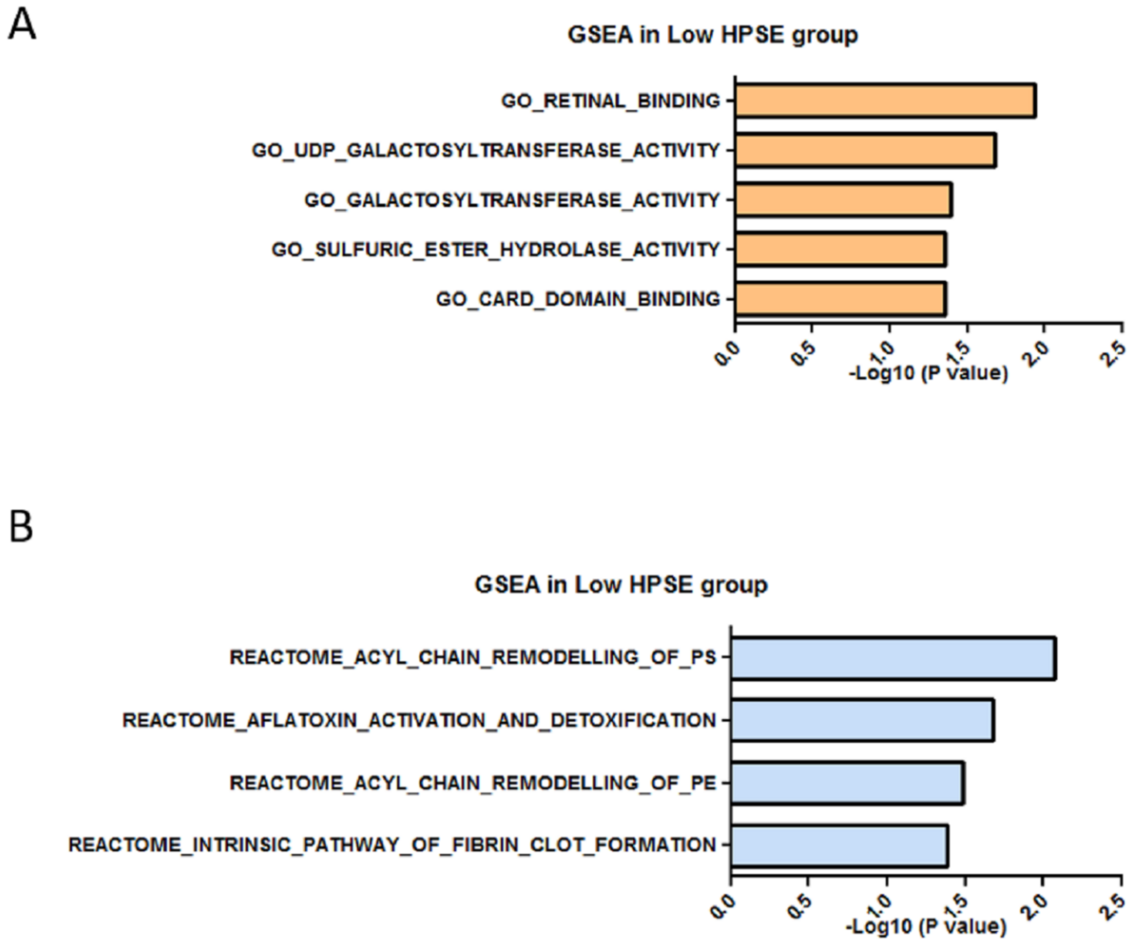

Figure S2: Top significant genes enriched in HPSE low patient group.

A-GSEA performed on HPSE low patient group and analyzed for set of genes related to biological process dataset. **B**-Top significant genes from Reactome gene sets enriched in HPSE low patient group performed by GSEA tool. Each signature was ranked by -log10 P value.
